# Multiple Bcl-2 family immunomodulators from vaccinia virus regulate MAPK/AP-1 activation

**DOI:** 10.1099/jgv.0.000525

**Published:** 2016-09

**Authors:** Alice A. Torres, Jonas D. Albarnaz, Cláudio A. Bonjardim, Geoffrey L. Smith

**Affiliations:** ^1^​ Laboratório de Vírus, Departamento de Microbiologia, Instituto de Ciências Biológicas, Universidade Federal de Minas Gerais, Av. Antonio Carlos, 6627, Pampulha, CEP 31270-901, Belo Horizonte, MG, Brazil; ^2^​ Department of Pathology, University of Cambridge, Cambridge, UK

**Keywords:** Vaccinia virus, immune evasion, NF-kB, MAP kinase, AP-1, Bcl-2 family

## Abstract

Vaccinia virus (VACV) is a poxvirus and encodes many proteins that modify the host cell metabolism or inhibit the host response to infection. For instance, it is known that VACV infection can activate the mitogen-activated protein kinase (MAPK)/activator protein 1 (AP-1) pathway and inhibit activation of the pro-inflammatory transcription factor NF-κB. Since NF-κB and MAPK/AP-1 share common upstream activators we investigated whether six different VACV Bcl-2-like NF-κB inhibitors can also influence MAPK/AP-1 activation. Data presented show that proteins A52, B14 and K7 each contribute to AP-1 activation during VACV infection, and when expressed individually outwith infection. B14 induced the greatest stimulation of AP-1 and further investigation showed B14 activated mainly the MAPKs ERK (extracellular signal-regulated kinase) and JNK (Jun N-terminal kinase), and their substrate c-Jun (a component of AP-1). These data indicate that the same viral protein can have different effects on distinct signalling pathways, in blocking NF-κB activation whilst leading to MAPK/AP-1 activation.

Viruses subvert cellular biochemistry and inhibit host defence mechanisms to facilitate their replication and spread (McFadden, 2005). Vaccinia virus (VACV) is the prototypic poxvirus and a large dsDNA virus that replicates in the cytoplasm. VACV infection triggers activation of the MAPKs (mitogen-activated protein kinases) ERK (extracellular signal-regulated kinase) and JNK (Jun N-terminal kinase) that are subverted to support replication and spread of the virus (de Magalhães *et al.*, 2001; Andrade *et al.*, 2004; Silva *et al.*, 2006; Pereira *et al.*, 2012). MAPKs are activated by many different stimuli, such as growth factors, stress and cytokines, and can generate several biological responses within the cell (Kyriakis & Avruch, 2012). One of the most important MAPK substrates is the transcriptional factor activator protein 1 (AP-1) (Whitmarsh & Davis, 1996). AP-1 is composed of dimers of basic region-leucine zipper (bZIP) proteins of the Jun (c-Jun, JunB and JunD), Fos (c-Fos, FosB, Fra1 and Fra2), activating transcription factor (ATF) (ATF2, ATF3/LRF1, B-ATF, JDP1 and JDP2), and musculoaponeurotic fibrosarcoma (Maf) (c-Maf, MafB, MafA, MafG/F/K and Nrl) families (Meng & Xia, 2011). Although it is known that VACV triggers AP-1 activation early after infection (de Magalhães *et al.*, 2001) the mechanism by which this occurs is not understood.

MAPK pathways interact with other signalling pathways, fine-tuning the appropriate cell response by integrating different extracellular and intracellular signals. Among those pathways, the signalling events leading to activation of transcription factor NF-κB share upstream activators with the MAPKs (Arthur & Ley, 2013). NF-κB is an important factor in inducing the immune response and VACV encodes at least ten intracellular proteins that inhibit activation of NF-κB (Sumner *et al.*, 2014). These proteins are all expressed early during infection and inhibit NF-κB activation at different steps in the signalling pathway (Smith *et al.*, 2013). In each case studied these proteins also affect virulence and their deletion causes attenuation (Smith *et al.*, 2013). Six of these inhibitors, A46, A49, A52, B14, K7 and N1, have had their structure solved and are all Bcl-2 family members (Cooray *et al.*, 2007; Graham *et al*, 2008; Kalverda *et al.*, 2009; Oda *et al*. 2009; Fedosyuk *et al.*, 2014; Neidel *et al.*, 2015). In this study we have examined if these Bcl-2 family inhibitors of NF-κB also modulate MAPK/AP-1.

To determine whether VACV proteins A46, A49, A52, B14, K7 and N1 could interfere with AP-1 transcriptional activity, AP-1 reporter gene assays were undertaken in HeLa and HEK293T cells. The AP-1 reporter plasmid (AP-1 Luc), encoding firefly luciferase under the control of consensus AP-1 binding element repeats [(TGACTAA)_7_], was a gift of Andrew Bowie (Trinity College Dublin) and the renilla luciferase control (TK-Ren) plasmid was from Promega. HEK293T (human embryonic kidney cell line) cells were maintained in Dulbecco’s modified Eagle’s medium (Gibco) supplemented with 10 % heat-treated (56 °C, 1 h) FBS (Biosera) and penicillin/streptomycin (P/S) (100 U ml^−1^ and 100 µg ml^−1^, respectively). HeLa (human cervical carcinoma) cells were maintained in minimum essential medium (Gibco) supplemented with 10 % FBS, non-essential amino acids (Sigma) and P/S. Human codon-optimized and Flag-tagged or TAP-tagged (containing Flag epitopes) versions of ORFs A46, A52, B14, K7 and N1, or non-codon optimized A49, were cloned into pcDNA4.1-TO (Invitrogen) and transfected (100 ng per well) into cells in triplicate with 150 ng AP-1-Luc and 10 ng TK-Ren plasmids per well, using TransIT-LT1 Transfection Reagent (Mirus Bio LLC) according to the manufacturer’s instructions. Twenty-four hours post-transfection, the cells were stimulated with phorbol 12-myristate 13-acetate (PMA; Sigma) (10 ng ml^−1^) for 24 h to activate AP-1. Cells were then harvested in passive lysis buffer (Promega), and the firefly and renilla luciferase activities were measured using a FLUOstar luminometer (BMG). The firefly luciferase activity in each sample was normalized to the renilla luciferase activity. Protein expression was confirmed by immunoblotting using rabbit anti-Flag (Sigma) and mouse anti-α-tubulin primary antibodies (Millipore), and goat anti-mouse or rabbit IRdye 800CW infrared dye secondary antibodies. Membranes were imaged using an Odyssey infrared imager (LI-COR Biosciences).

Fig. 1(a, b) shows that proteins A52, B14 and K7 in HeLa cells, and proteins A52 and B14 in HEK293T cells, caused a significant increase in the levels of AP-1 reporter activity induced by PMA when compared to empty vector (EV). Furthermore, B14 also induced AP-1 in the absence of PMA stimulation. In contrast, A49 expression led to a reduction of the AP-1 reporter activity, and proteins A46 and N1 did not alter AP-1 activity when compared to the EV control. The panels below each graph show that the expression levels of the different proteins were similar, except A49, which was expressed at much lower levels, especially in HeLa cells. Previously, it was reported that A46 inhibits Toll-like receptor/IL-1-stimulated MAPKs and NF-κB activation, whereas A52 can activate p38 MAPK and JNK activity, but whether these Bcl-2-like proteins can also induce AP-1 was not demonstrated (Bowie *et al.*, 2000;  2005, Stack *et al.*, 2013; Stack & Bowie, 2012; Maloney *et al.*, 2005; Keating *et al.*, 2007). B14 was also reported to increase PMA-stimulated AP-1 activity (Chen *et al.*, 2008). 

**Fig. 1.**
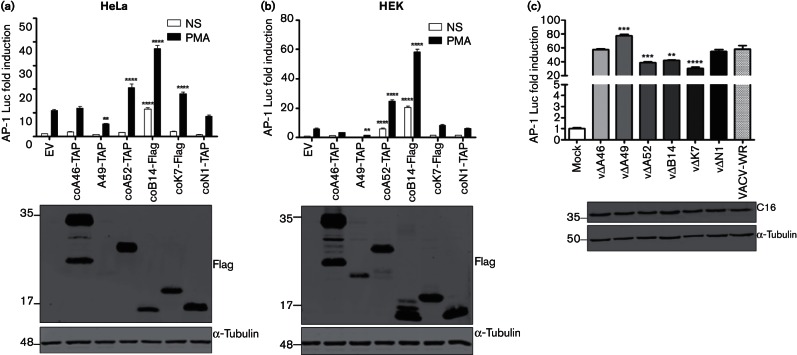
VACV proteins A52, B14 and K7 induce AP-1. HeLa (a) and HEK293T (b) cells were co-transfected in triplicate with an AP-1 luciferase reporter, a renilla luciferase internal control and expression vectors for the proteins shown. Twenty-four hours post-transfection, the cells were stimulated for 24 h with PMA or left non-stimulated (NS). (c) AP-1 reporter gene assay in HeLa cells mock-infected or infected for 24 h with VACV-WR (wild-type) or deletion viruses (10 p.f.u. per cell). Luminescence was measured and normalized to that of EV non-stimulated or of mock-infected cells to give the fold induction. Data are shown as the mean±sd and are representative of three experiments. Statistical analysis was performed by unpaired Student’s *t*-test (**P<0.01, ***P<0.001, ****P<0.0001) in comparison to EV control, either non-stimulated (NS) or PMA-stimulated (a and b), or VACV-WR (c). The panels below each graph show protein expression controls by immunoblotting; molecular masses (in kDa) are indicated on the left.

Next, the influence of A52, B14 and K7 on AP-1 activation was also investigated during VACV infection. HeLa cells were transfected with the reporter plasmids and then were either mock-infected or infected with VACV wild-type (WR strain; VACV-WR) or mutants lacking gene *A46R* (vΔA46, Stack *et al.*, 2005), *A49R* (vΔA49, Mansur *et al.*, 2013), *A52R* (vΔA52, Harte *et al.*, 2003), *B14R* (vΔB14, Chen *et al*. 2006), *K7R* (vΔK7, Benfield *et al.*, 2013) or *N1L* (vΔN1, Bartlett *et al.*, 2002) for 24 h (10 p.f.u. per cell). Infection was monitored by immunoblotting as described with rabbit anti-C16 (Fahy *et al*. 2008). VACV infection induced AP-1 reporter activity when compared to mock-infected cells (Fig. 1c), as demonstrated previously (de Magalhães *et al.*, 2001). However, the degree of activation was reduced in the absence of A52, B14 or K7 proteins (Fig. 1c) and enhanced by lack of A49, and these results are consistent with ectopic expression of these proteins (Fig. 1a, b). Lastly, loss of A46 and N1 did not affect AP-1 activation during infection. Immunoblotting for VACV protein C16 showed that the infection was comparable among the different viruses (Fig. 1c).

Further investigation of AP-1/MAPK activation during VACV infection was undertaken with protein B14 because it exerted the greatest increase in AP-1 activity. First, B14 was shown to increase AP-1 expression in a dose-dependent manner in both non-stimulated and PMA-stimulated cells (Fig. 2a). Similarly, AP-1 reporter activity was increased during infection proportionate to the multiplicity of infection and with time post-infection, and vΔB14 induced consistently lower AP-1 activity than VACV-WR (Fig. 2b). The level of infection in these cells was confirmed by immunoblotting for the VACV protein C6 (Unterholzner *et al.*, 2011).

**Fig. 2.**
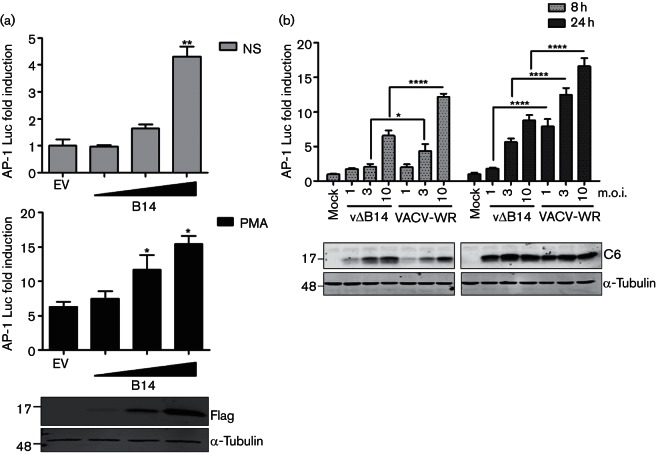
B14 stimulates AP-1 in a dose-dependent manner following transfection and infection. (a) HeLa cells were co-transfected in triplicate with an AP-1 luciferase reporter, a plasmid expressing renilla luciferase and increasing amounts of the B14 vector or the empty vector (EV). Twenty-four hours later, the cells were stimulated for 24 h with PMA (bottom graph) or left non-stimulated (NS) (top graph). (b) Reporter gene assay in HeLa cells mock-infected or infected for 8 or 24 h with VACV-WR (wild-type) or B14 deletion (vΔB14) viruses, using the indicated multiplicity of infection (m.o.i.). The luminescence of each sample was measured and normalized to that of EV non-stimulated(NS) (a) or mock-infected cells (b) to give the fold induction. Data are shown as the mean±sd and are representative of three experiments. Statistical analysis was by unpaired Student’s *t*-test (*P<0.05, **P<0.01, ****P<0.0001) in comparison to EV control, either non-stimulated or PMA-stimulated (a) or between vΔB14 and VACV-WR (b). The panels below each graph show protein expression controls by immunoblotting; molecular masses (in kDa) are indicated on the left.

AP-1 is the main substrate of the different MAPK pathways, and so which MAPK was activated by B14 was investigated next. Firstly, AP-1 reporter activity induced by B14 was measured in the presence of specific MAPK inhibitors. HeLa cells were co-transfected with the B14 expression vector and the reporter plasmids, and 24 h later, cells were treated with 15 µM U0126 (inhibitor of MEK1/2, the upstream activator of ERK), 10 µM SB203580 (p38α/β MAPK inhibitor), 4 µM JNK inhibitor VIII (JNK1/2 inhibitor) or with DMSO, and were stimulated with PMA or left non-stimulated. In the presence of MEK/ERK and JNK inhibitors, the induction of AP-1 by B14 was decreased significantly (Fig. 3a). In contrast, p38 MAPK inhibition had the opposite effect on the levels of AP-1 activity induced by B14 alone and no effect on the enhancement of PMA-stimulated AP-1 activity by B14. This result suggests that B14 is modulating predominantly ERK and JNK to stimulate AP-1 activity. To confirm this suggestion, the phosphorylation levels of the MAPK proteins and the transcription factor c-Jun were measured when B14 was expressed ectopically or during infection with VACV-WR and vΔB14 in HeLa cells. Cells were washed twice on ice with ice-cold PBS, and scrapped into cell lysis buffer (50 mM Tris pH 8, 150 mM NaCl, 1 mM EDTA, 10 % glycerol, 1 % Triton X-100 and 0.05 % NP-40) supplemented with protease and phosphatase inhibitors (Roche). Cell lysates were subjected to SDS-PAGE and immunoblotting using the following antibodies from Cell Signaling Technology: phospho-ERK1/2 (Thr202/Tyr204, #9101), phospho-p38 MAPK (Thr180/Tyr182, #9211), phospho-JNK1/2 (Thr183/Tyr185, #9251), and phospho-c-Jun (Ser63, #9261). Infection was confirmed by immunoblotting with mouse mAb against VACV protein D8 (Parkinson & Smith, 1994) and rabbit anti-B14 serum (Chen *et al.*, 2006).

**Fig. 3.**
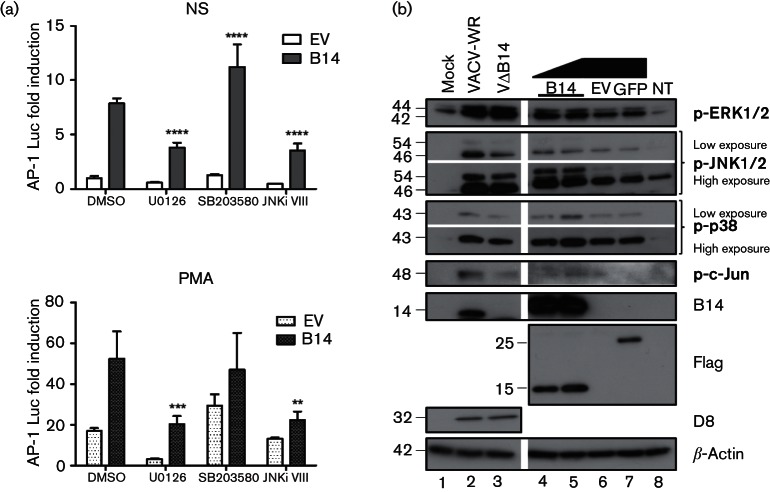
Contribution of B14 to MAPK activation (a) HeLa cells were co-transfected in triplicate with an AP-1 luciferase reporter, a renilla luciferase reporter and B14 vectors. After 24 h, cells were treated with U0126 (MEK/ERK inhibitor), SB203580 (p38 MAPK inhibitor), JNK inhibitor VIII (JNK1/2 inhibitor) or DMSO, and stimulated for 24 h with PMA (10 ng ml^−1^) or left non-stimulated (NS). The luminescence of each sample was measured and normalized to that of the non-stimulated control. Data are shown as the mean±sd and are representative of three experiments. Statistical analysis was by Student’s *t*-test (**P<0.01, ***P<0.001, ****P<0.0001). (b) HeLa cells were mock-infected or infected with VACV-WR or vΔB14 (lanes 1, 2 and 3, respectively) for 12 h (5 p.f.u. per cell). In parallel, cells were transfected with the B14 (1 or 2 µg), GFP or empty (EV) vectors or left non-transfected (NT) for 24 h (lanes 4, 5, 7, 6 and 8, respectively). Cells were harvested and lysates were subjected to immunoblotting for the proteins shown; molecular masses (in kDa) are indicated on the left.

Even though inhibition of the MEK/ERK pathway had resulted in a significant decrease in AP-1 activation by B14, there was no difference in the levels of activated ERK1/2 in the absence of B14 during the infection (Fig. 3b, lanes 2 and 3). This might be due the existence of other known proteins encoded by VACV that are able to induce ERK activation, such as VGF and O1 (Andrade *et al.*, 2004; Schweneker *et al.*, 2012). However, when B14 is expressed alone (Fig. 3b, lanes 4 and 5), an increase of activated ERK1/2 was seen compared with EV and GFP (Fig. 3b, lanes 6 and 7) even when the amount of B14 plasmid was reduced twofold (Fig. 3b, lane 4). There was a slight reduction in the phosphorylation of p38 MAPK during infection with vΔB14 (Fig. 3b, lanes 2 and 3) and a small increase in its phosphorylation in the presence of B14 only when 2 µg of plasmid were used (Fig. 3b, lane 5). In contrast, a decrease in JNK1/2 and c-Jun phosphorylation was observed in the absence of B14 during infection (Fig. 3b, lanes 2 and 3), and consistent with this observation, there was an increase in activated JNK1/2 and c-Jun in cells transfected with the B14 plasmid (Fig. 3b, lanes 4 and 5) compared with the controls (Fig. 3b, lanes 6 and 7).

Taken together, data presented show that VACV proteins A52, B14 and K7, which are all NF-κB inhibitors, contribute to the activation of AP-1 not only when expressed alone but also during infection, while A49 has the opposite effect. The fact that the lack of only one protein resulted in decreased levels of AP-1 reporter activity in infected cells suggests that these proteins do not have redundancy in their mechanisms of AP-1 activation. Other viruses also induce AP-1 during infection. For instance, Epstein–Barr virus (EBV) encodes several proteins that modulate the MAPK pathways and contribute to AP-1 activation and viral reactivation from latency. EBV protein BRLF1 modulates all three MAPK pathways (Adamson *et al.*, 2000), while EBV protein BZLF1 activates p38 and JNK MAPKs (Adamson *et al.*, 2000; Lee *et al.*, 2008) thereby inducing AP-1 activity. More recently, it was demonstrated that EBV protein BGLF2 also activates AP-1 by regulating the p38 MAPK (Liu & Cohen, 2016).

The role of B14 in AP-1 activation was seen clearly during infection by comparing vΔB14 and VACV-WR and this correlated with activation of JNK, which is the main kinase responsible for phosphorylation of the transcription factor c-Jun, the major transcriptional activator of AP-1 (Meng & Xia, 2011). The removal of B14 did not inhibit JNK activation completely, suggesting the existence of additional VACV proteins that activate this pathway. Consistent with this, expression of VACV B1 kinase upregulated activated JNK and c-Jun, but that was not demonstrated in the context of infection (Santos *et al.*, 2006).

Activation of MEK/ERK during infection by multiple VACV proteins including the Bcl-2 proteins described here, and VGF and protein O1 described previously, aids VACV replication and suppression of cell death (de Magalhães *et al.*, 2001; Andrade *et al.*, 2004; Postigo *et al.*, 2009). JNK activation during VACV infection is important for regulation of cytoskeleton reorganisation required for viral spread (Pereira *et al.*, 2012). Taken together these observations suggest that A52, possibly via p38 MAPK (Maloney *et al.*, 2005), B14, mainly via JNK/c-Jun, and K7 are increasing AP-1 activity to promote VACV multiplication and spread, in addition to their inhibition of NF-κB activity.

In summary, these findings show that VACV Bcl-2 family members A52, B14 and K7 modulate AP-1 activity during infection in addition to their known function as inhibitors of activation of NF-κB and IRF-3 (for K7), illustrating the multi-functional nature of these small alpha-helical proteins. This may explain in part why removal of these NF-κB inhibitors individually from VACV gives an *in vivo* phenotype despite the presence of multiple other inhibitors of this pathway.
